# O.4.6-9 Physical activity during school time in Denmark: an accelerometer-based study

**DOI:** 10.1093/eurpub/ckad133.228

**Published:** 2023-09-11

**Authors:** Nanna Holt Jessen, Sebastian Skejø, Lasse Ziska, Rasmus Østergaard Nielsen

**Affiliations:** Research Unit for General Practice, Aarhus, Denmark; Research Unit for General Practice, Aarhus, Denmark; Section for IT and Digitalisation, Syddjurs Municipality, Denmark; Research Unit for General Practice, Aarhus, Denmark; Department of Public Health, Aarhus University, Aarhus, Denmark; n.holt@ph.au.dk

## Abstract

**Purpose:**

The Danish Primary School Act stipulates that each school day must include an average of 45 minutes of physical activity (PA) for pupils. However, the law does not specify the recommended level of intensity or whether breaks must be included. This study aimed to investigate the amount and intensity of PA during school time among 4th grade pupils in Danish public schools. An additional aim was to investigate if differences between low-intensity PA and moderate-to-vigorous PA (MVPA) exist. Thereby, this study can contribute with new perspectives on statutory PA during school time.

**Methods:**

The study was conducted in Syddjurs Municipality, Denmark, in the spring of 2021. All 10 schools in the municipality chose to participate. Pupils from 4th grade carried an accelerometer on the wrist of their non-dominant arm during school time. Following a familiarization period of 10 days, data was collected for another 10 school days (Monday to Friday in two consecutive weeks). Complete days were defined as days where the pupil wore the accelerometer during the entire school day.

**Results:**

A total of 411 pupils were included in the study. The analysis comprised 381 pupils with 2055 complete days. Low intensity PA for at least 45 minutes during the entire school day (including breaks) was performed by 99.6% (95%CI:99.2%;99.8%) of the pupils. Correspondingly for MVPA, 33.6% (95%CI:31.5%;35.7%) of the pupils had at least 45 minutes of MVPA during the entire school day. During lessons only (excluding breaks), 97.9% (95%CI:97.2;98.4%) of the pupils had at least 45 minutes of low intensity PA. The corresponding proportion of pupils performing MVPA during lessons was 16.8% (95%CI:15.2%;18.5%).

**Conclusions:**

Most pupils in 4th grade accomplished to perform PA of low intensity during school time, while one third of the pupils performed at least 45 minutes of MVPA during the entire school time, although MVPA is known to promote physical, mental and social health. If the law is intended to promote the health of school children, the recommended level of intensity need to be further specified.

**Support/Funding Source:**

The project was funded by Nordic Innovation.

